# Daratumumab therapy for post-HSCT immune-mediated cytopenia: experiences from two pediatric cases and review of literature

**DOI:** 10.1186/s40348-021-00114-y

**Published:** 2021-04-29

**Authors:** Lina Driouk, Robert Schmitt, Anke Peters, Sabine Heine, Hermann Josef Girschick, Brigitte Strahm, Charlotte M. Niemeyer, Carsten Speckmann

**Affiliations:** 1 Department of Pediatrics and Adolescent Medicine, Division of Pediatric Hematology and Oncology, Medical Centre, Faculty of Medicine, University of Freiburg, Mathildenstr. 1, 79106 Freiburg, Germany; 2 Department of Pediatric Hematology and Oncology, Saarland University Homburg, Homburg, Germany; 3 Vivantes Klinikum Friedrichshain, Children’s Hospital, Berlin, Germany; 4 German Cancer Consortium (DKTK), Freiburg, Germany; 5 German Cancer Research Center (DKFZ), Heidelberg, Germany; 6 Center for Chronic Immunodeficiency, Medical Center, Faculty of Medicine, University of Freiburg, Freiburg, Germany

**Keywords:** Daratumumab, Refractory cytopenia, Immune cytopenia, HSCT, AIT, AIHA

## Abstract

**Background:**

Immune-mediated cytopenias (AIC) are challenging complications following allogeneic hematopoietic stem cell transplantation (HSCT). While broad-acting immunosuppressive agents like corticosteroids are often standard of care, several novel therapies which target specific immunological pathways have recently been developed and provide hope for patients with steroid-refractory courses and may limit long-term toxicity. The successful off-label use of the plasma cell depleting anti-CD38 antibody daratumumab was published in several case reports, suggesting efficacy, i.e., in patients with antibody-mediated AIC refractory to previous B cell depletion. We want to share our experience with two children, whom we treated with daratumumab, including one fatal course with uncontrolled disease. Given the absence of substantial data from HSCT registries or prospective trials, we furthermore provide a critical review of the literature on daratumumab treatment of AIC.

**Case presentations:**

Patient 1 (P1), an 11-year-old girl with lipopolysaccharide-responsive and beige-like anchor protein (LRBA) deficiency who developed immune-mediated thrombocytopenia (AIT) from day +58 after HSCT, showed a complete response to daratumumab after the fourth of six total daratumumab doses. She remains transfusion independent for over a year of follow-up. Previously, her thrombocytopenia was refractory to corticosteroids, rituximab, intravenous immunoglobulins (IVIG), eltrombopag, cyclosporine A, and sirolimus. Patient 2 (P2), a 6-year-old boy with CD40 ligand (CD40L) deficiency, developed both AIT and hemolytic anemia (AIHA) after HSCT on days +58 and +83, respectively, and was also treated with daratumumab after being previously refractory to prednisolone, rituximab, and IVIG. Yet, he did neither respond to daratumumab nor the concomitantly administered methyprednisolone pulse, plasmapheresis, and eculizumab and succumbed due to refractory disease.

**Conclusion:**

Reviewing the literature on the use of daratumumab for refractory AIC post-HSCT, we consider daratumumab a promising agent for this life-threatening disorder: ten of the twelve patients reached transfusion independency in the literature. However, treatment failures are likely to be underreported. Thus, controlled trials are needed to explore the safety and efficacy of daratumumab in this rare post-HSCT complication.

## Case presentations

### Patient 1

Patient 1 is an 11-year-old girl from non-consanguineous Caucasian parents with lipopolysaccharide-responsive and beige-like anchor protein (LRBA) deficiency who underwent an allogeneic HSCT for the treatment of refractory immune dysregulation.

LRBA deficiency is a rare primary immunodeficiency characterized by the combination of a pathological susceptibility to infection and autoimmune phenomena including inflammatory bowel disease, diabetes mellitus, arthritis, autoimmune cytopenia, and interstitial lung disease. LRBA is an important regulator of the cytotoxic T-lymphocyte-associated protein 4 (CTLA-4), which plays a major role in the modulation of immune responses of T cells and other immune cells [1]. Patient 1 is compound heterozygous for two deletions—a paternally inherited deletion of exons 3–48 and a small maternally inherited deletion in exon 23. In the western blot, LRBA protein was undetectable, demonstrating that both mutations are disease-causing (data not shown). One of the patient’s siblings also had LRBA deficiency and succumbed due to uncontrollable autoimmune cytopenia at the age of 4.

P1 developed various clinical problems in the context of LRBA deficiency. The leading clinical problem had been infant-onset autoimmune enteropathy, which resulted in failure to thrive and necessity of long-term partially and finally complete parenteral nutrition and additional tube feeding since infancy. Insulin-dependent diabetes mellitus with repeated crisis-like derailments was first diagnosed at the age of 1.5 years. Since the age of 4, P1 presented with oligoarthritis of the lower extremities. In contrast to her deceased LRBA-deficient brother, she had no history of autoimmune cytopenia before HSCT was performed. Moreover, no pathologic susceptibility to infections had been noted before HSCT, and B cell differentiation was normal without the need for immunoglobulin substitution. Because of her multiple autoimmune manifestations, P1 received long-term immunosuppressive therapy with azathioprine (since 1.5 year of age), sirolimus (since 3 years of age), intermittent systemic steroid therapies during autoimmune flare-ups, abatacept (for 6 months at the age of 6 years; discontinued due to associated fever episodes), hydroxychloroquine since the age of 8 years, and intermittent anti-inflammatory therapy with ibuprofen for knee joint arthritis.

The indication for allogeneic HSCT was based on the insufficient control of autoimmunity despite multimodal immunosuppressive therapy, persistent enteropathy, and failure to thrive despite parenteral nutrition and the expected aggravating natural course of the disease.

Following a myeloablative preparative regimen consisting of thiotepa (1 × 8mg/kg), treosulfan (3 × 14g/m^2^), fludarabine (4 × 40mg/m^2^), and anti-thymocyte globulin (3 × 15 mg/kg), the patient was grafted with bone marrow from a 10/10 HLA-matched unrelated female donor. She received 7.32 × 10^8^ nucleated cells per kilogram of body weight, changing blood type from B rhesus positive to O rhesus positive. She experienced early complete engraftment with a leukocyte, neutrophil, and platelet engraftment on days +18, +20, and +24, respectively. Following graft-versus-host disease (GvHD) prophylaxis with methotrexate and cyclosporine A, we observed no signs of GvHD. Besides transient grade 2 mucositis, the girl had an uneventful post-transplant course. The chimerisms on days +29 and +53 in the peripheral blood showed full donor origin.

Between day +58 and day +61 after HSCT, our patient’s platelet count dropped from 142.000 to 5.000/μl, clinically compatible with an immune-mediated thrombocytopenia. We excluded thrombotic microangiopathy as a potential cause of the thrombocytopenia (normal LDH, no fragmented red blood cells, continuously controlled hypertension, no proteinuria) [2] and measured a normal ADAMTS13 level and activity, no antibodies against ADAMTS13, and a stable creatinine. We performed platelet-associated antibody testing, which revealed the presence of free anti-platelet antibodies in the patient’s serum, mostly reactive against the glycoprotein complex GIIb/IIIa. Testing for platelet HLA antibodies was negative.

In the first weeks of thrombocytopenia, the only bleeding signs were skin hematomas, petechia, and mild oral mucosal bleeding. Gradually, P1 started experiencing recurrent episodes of Hb-relevant bleeding (severe epistaxis and gastrointestinal bleeding), requiring multiple packed red blood cell (PRBC) transfusions. Overall, since the diagnosis of the immune-mediated thrombocytopenia, P1 received 163 platelet and 18 PRBC transfusions.

**Fig. 1:**
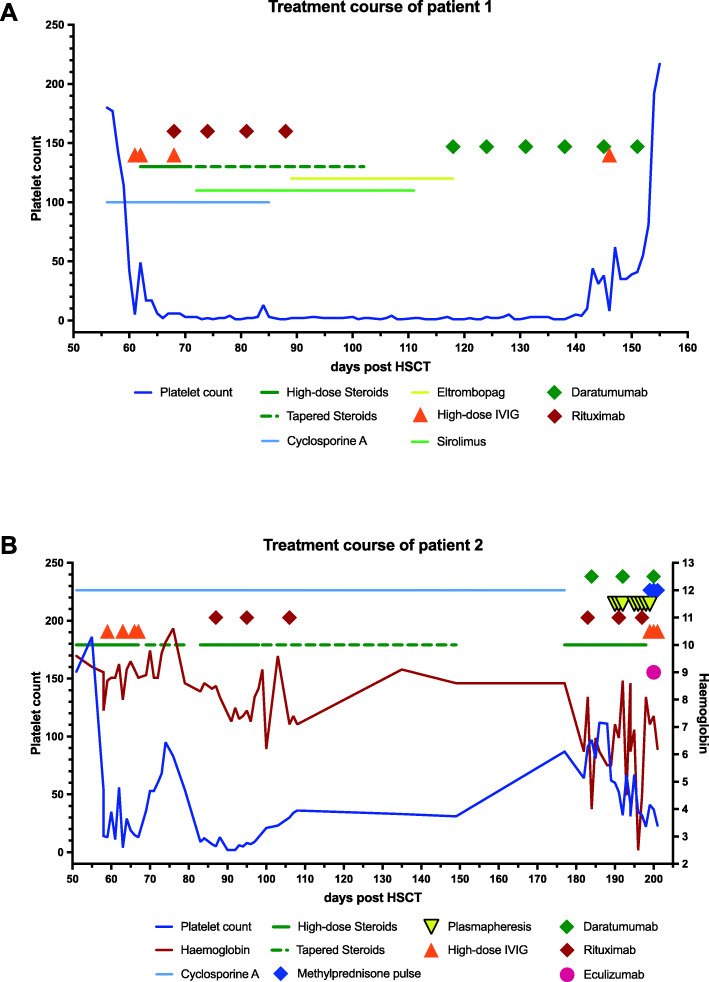
Platelet count and therapy for autoimmune cytopenia following HSCT in patient 1 (**a**) and patient 2 (**b**)

We initiated treatment with high-dose corticosteroids (initially 2 mg/kg/day) and 4 doses of intravenous immunoglobulin (1 g/kg) from day +61 onward (Fig. 1a). Additionally, P1 received 4 doses of rituximab (375 mg/m^2^) and eltrombopag 50 to 75 mg daily, all without significant impact on her platelet count. Cyclosporine A (trough level 150–200 ng/ml), primarily administered for GvHD prophylaxis, was switched to sirolimus (trough levels 8–12 ng/ml), also without any significant response.

Given the persistence of severe thrombocytopenia despite B cell depletion with rituximab, we hypothesized that plasma cells are the likely source of the anti-platelet antibodies and initiated a plasma cell depleting therapy with daratumumab in week 8 of P1’s AIT. We administered the anti-CD38 antibody in a dose of 16 mg/kg per week i.v. The first infusion caused nasal congestion and bronchial obstruction, whereas the next infusions were well tolerated. Twenty-five days after the first dose of daratumumab and after a total of 4 administrations, the girl’s platelet count rose rapidly and the bleeding stopped. The patient’s platelet count reached 55.000/μl on day +152 after HSCT, when she was discharged, and normalized to 192.000/μl on day +159, 41 days after the first dose of daratumumab. Nine months after the cessation of treatment with daratumumab, the girl is clinically well with normalized platelet counts. One year after HSCT, we continuously see full donor chimerism and no symptoms of LRBA deficiency besides her pre-HSCT existing type I diabetes, which is well controlled. P1 experiences a gradual B cell recovery, albeit still relying on substitution of immunoglobulins.

### Patient 2

Patient 2 was a 6-year-old boy from non-consanguineous Caucasian parents with CD40L deficiency who underwent allogeneic HSCT because of the expected natural history of CD40L deficiency with severe opportunistic infections and non-reversible organ damage.

CD40L deficiency, also referred to as X-linked hyper IgM syndrome, is a rare primary immunodeficiency characterized by an immunoglobulin class switch defect from IgM to IgG and a defect in T cell activation. CD40L acts as a costimulatory molecule which induces a variety of effects in the adaptive immune system after binding to its receptors, the most important one of which is CD40 on antigen-presenting cells. CD40L-deficient patients suffer from recurrent bacterial infections from early childhood as well as opportunistic infections, non-infectious diarrhea, and liver disease [3]. HSCT outcome and long-term quality of life are more favorable, if performed early and in the absence of previous opportunistic infection and/or end-organ damage [4].

P2 was diagnosed with CD40L deficiency at the age of 4.5 years. He was hemizygous for a pathogenic mutation (c.686T>C; p.Phe229Ser) in the *CD40L* gene*.* The mother was identified as a carrier. Functional testing confirmed defective upregulation of CD40L on T cells after PMA stimulation in vitro (data not shown).

At age 1 he presented with a pronounced eyelid phlegmon for which he was hospitalized. Subsequently, he showed recurrent bilateral episodes of perforating otitis media. In the 3rd and 4th year of life, radiologically confirmed pneumonia with protracted recovery occurred. At the age of 3 years, P2 experienced an onset of transient diarrhea episodes, for which no infectious triggers could be identified and was therefore judged an immune dysregulatory manifestation of his disease. From age 5 onward, P2 received prophylactic treatment by intravenous immunoglobulin substitution and cotrimoxazole.

P2 underwent HSCT at the age of 6 years and was grafted with bone marrow (6.81 × 10^8^ nucleated cells per kilogram of body weight) from a 9/10 HLA-matched unrelated male donor, following the same conditioning regimen and GvHD prophylaxis as P1. He changed blood type from O rhesus negative to O rhesus positive. During the uneventful post-transplant course, besides grade 2 mucositis, there was timely hematologic recovery with leukocyte and neutrophil engraftment on day +22 and platelet engraftment on day +20. We observed no signs of acute GvHD. The chimerisms on days +30, +43, +62, +100, and +149 in the peripheral blood and on day +149 in the bone marrow aspirate showed full donor origin.

Between day +55 and day +58 after HSCT, P2’s platelet count instantly dropped from 186.000 to 14.000/μl, and he was diagnosed with post-transplant AIT. We initiated treatment with 2 mg/kg/day prednisolone, combined with four doses of IVIG (0.4g/kg/day), and continued GvHD prophylaxis with cyclosporine A. This regimen initially resulted in a transient recovery of P2’s platelet count (Fig. 1b).

During the following prednisolone tapering, the patient’s AIT relapsed on day +83 with a dropping platelet count to 9 × 10^3^/μl. In addition, P2 was then additionally diagnosed with first signs of AIHA, based on the signs of hemolysis in the laboratory analysis (hemoglobin 8.6 g/dl, reticulocytosis of 41‰, positive direct Coombs test). Further immunological studies revealed the presence of anti-E, heat, and cold autoantibodies as well as free platelet-reactive antibodies, in P2’s serum, providing immunological correlates of AIHA and AIT.

Since steroid treatment was refractory, we administered high-dose IVIG (1 g/kg/dose) in combination with rituximab (375 mg/m^2^/dose, a total of weekly 6 doses) starting day +87. Due to severe thrombocytopenia with platelet counts of 2–9 × 10^3^/μl, our patient received daily single-donor apheresis platelets.

With this regimen, P2 showed again signs of response on day +98 and reached a phase with a stable platelet count and less hemolysis during prednisolone tapering. Unfortunately, on day +177 our patient relapsed again with signs of increased hemolysis, yet a stable platelet count.

We increased prednisolone again to 2 mg/kg and extended our therapy to daratumumab (16 mg/kg/dose) on day +184 with the rationale to deplete antibody-producing plasma cells after an unavailing B cell depletion. Upon the first administration of daratumumab, P2 developed transient and uncomplicated bronchial obstruction and vomiting. The following doses on days +192 und +200 were tolerated well.

On day +190, P2 experienced another acute flare of autoimmune hemolyis. Given the rapid dynamics of the AIHA, we initiated additional antibody reduction by plasmapheresis, which was performed on days +190 to +192 and +195 to +199. The patient required daily PRBC transfusions.

Despite the high frequency of plasmapheresis, the elimination of RBC antibodies was not sufficient enough and starting from day +199 P2 developed signs of a hypovolemic shock and aggravated hemolysis with Hb levels <3 g/dl. He received a salvage therapy by methylprednisolone pulses (20 mg/kg/day for 3 days), three doses of IVIG (1 g/kg/day), and one dose of eculizumab (25 mg/kg). Without any response to this therapy, P2 deceased from cardiac arrest due to acute heart failure most likely related to pulmonary embolism.

## Review of literature

To our knowledge, eight previous reports presented data on ten patients [5–12]. A search for additional cases of AIC was conducted through PubMed, using the keyword daratumumab combined with HSCT, AIC, AIHA, hemolytic, Evans syndrome, ITP, thrombocytopenia, immune dysregulation, and autoimmunity (excluding multiple myeloma). The identified articles were checked for duplicate patients and cross-references.

Including our P1 and P2, four of twelve patients were diagnosed with pure red cell aplasia (PRCA), three with AIHA, two with AIT, and three with Evans syndrome (ES). Five patients were children (all <12 years of age) and seven adults (age 19–72 years, median 43). The primary disease leading to HSCT was acute leukemia (*n*=2), myelodysplastic syndrome (*n*=3), severe aplastic anemia (*n*=2), primary myelofibrosis (*n*=1), or a primary immunodeficiency disease (*n*=4). Six patients received peripheral blood stem cells (PBSC), three whole bone marrow, and in three cases the stem cell source was not reported. Five patients were transplanted from a 10/10 and three from a 9/10 unrelated donor. Three patients were transplanted from a family donor. The four patients with PRCA and one patient each with AIT and ES received an ABO mismatch graft. Chimerism was 100% donor in 6 and >90% in the other six patients. Excluding patients with PRCA, the onset of AIC was at a median of day +127 (range +58 to +359), and the first dose of daratumumab was administered at a median of 189 days (range 63–503 days) after AIC diagnosis. In patients with PRCA, the first daratumumab was given at days +206, +390, and +411, and at 2.5 years after HSCT. In all cases, daratumumab was administered once weekly at a dose of 16mg/kg. The median number of infusions was 6 (range 2–11). Patients had previously received two to nine different therapies, most commonly corticosteroids, rituximab, and bortezomib (Table 1). In nine patients, at least one concomitant treatment was given during daratumumab therapy. Ten patients reached transfusion independency, for seven of whom a normalization of the blood count was reported. One patient showed a transient partial response, defined as a decreased transfusion frequency, and one patient did not respond to daratumumab. The response was noted after one to six weekly doses of daratumumab. While outcome measures were defined differently and not all reports state whether the patient normalized the blood counts transfusion independency was always scored as a response. The median follow-up time after daratumumab treatment among the ten patients who reached transfusion independency was 8 months (range 4–16 months), and all patients were alive without severe long-term toxicities. Therapy with IVIG substituion was specified for six patients, out of whom four still were treated with IVIGs at the time of reporting or death.

**Table 1:**
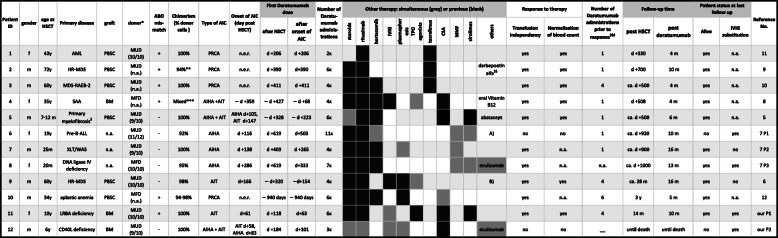
Clinical summary of twelve patients with autoimmune cytopenia following stem cell transplantation who received therapy with daratumumab

*Abbreviations*: *AIC* Immune-mediated cytopenia (allo- or autoimmune cytopenia), *AIHA* Allo- or autoimmune hemolytic anaemia, *AIT* Allo- oder autoimmune thrombocytopenia, *CD40L* CD40 ligand, also called CD154, *d* Day, *HLA* Human leukocyte antigen, *HR-MDS* High risk myelodysplastic syndrome, *HSCT* Hematopoietic stem cell transplantation, *IVIG* i.v. immunoglobulin G, *LRBA* Lipopolysaccharide-responsive and beige-like anchor protein, *m* Month, *MDS-RAEB-2* Myelodysplastic syndrome – refractory anemia with blast excess 2 (in transformation), *n.a.* Not available, *n.e.r.* No erythrocyte recovery, *PBSC* Peripheral blood stem cells, *PRCA* Pure red cell aplasia, *FD* Family donor, *SAA* Severe aplastic anemia, *UD* Unrelated donor, *XLT/WAS* X-linked thrombocytopenia / Wiskott Aldrich syndrome, *y* Year

^*^Donor type: matched unrelated donor (MUD) or matched family donor (MFD). HLA match was indicated where available, e.g. 10/10. If not further specified by the authors marked with n.a

^**^94% donor leukocytes with a 57% T-cell subset

^***^Granulocytes 92-100%, T-cells 6-48%

^$^VPS45 protein deficiency

^$$^no information on simultaneous therapies

^$$$^ response as defined by the authors

A) alemtuzumab, ibrutinib, Cy, ATG (alemtuzumab not concomitant) B) vincristine, splenectomy, danazol (just danazol concomintant)

## Discussion and conclusions

AIC is a challenging complication following allogeneic HSCT, mostly occurring in patients with a non-malignant primary disease [13, 14]. Other risk factors for AIC are age <15 years, an unrelated donor, human leukocyte antigen (HLA) mismatch, a stem cell graft other than bone marrow, a conditioning regimen with alemtuzumab, cytomegalovirus (CMV) reactivation, and chronic graft-versus-host disease (GvHD) [13–16]. AIC is observed in 2.5–9.4% of pediatric or adult patients [15–19], with the largest cumulative incidence of 9.4% being reported in a cohort of 502 children with primary immunodeficiency who underwent HSCT [19]. The phenomenon presents as AIHA, AIT, PRCA, defined as the absence of erythrocyte recovery after HSCT, immune-mediated neutropenia, or a combination of these (Evans syndrome; ES) [17]. Not only the cytopenia itself poses a potentially life-threatening risk for the patients, but also the toxic side effects and the likelihood of severe infections deriving from multimodal immunosuppressive therapy.

Besides the fusion protein abatacept blocking T cell activation via CTLA-4, and the anti-C5 antibody eculizumab preventing terminal complement activation, daratumumab, an anti-CD38 antibody targeting plasma cells, crystalizes out as a promising treatment option [15] for refractory post-transplant AIC. Daratumumab was designed for the treatment of multiple myeloma and FDA approved in 2015. With the rationale that antibody-producing CD38+ plasma cells—either autologous or allogeneic—are a potential cause of AIC, we treated two children with antibody-mediated refractory cytopenia following HSCT with daratumumab [20, 21].

The data presented demonstrate that daratumumab treatment of refractory post-HSCT AIC can be effective and life-saving. Yet, single-case reports of variable detail have a low evidence level and it is likely that treatment failures are underreported [22]. Long-term responses, the incidence of infections, and potential long-term toxicity such as the impairment of the humoral immunity cannot be evaluated because of generally short follow-up. Treatment of AICs with daratumumab warrants caution given the lack of long-term experience, the off-label use, and the fact that daratumumab may not always be the best suitable therapy since immune-mediated cytopenias can derive from different forms of immune dysregulation [23], not all of which involve plasma cells. Despite being usually reported as a well-tolerated substance, daratumumab poses a significant risk for infectious complications [24], which is not well evaluated in children. Thus, it should not be underestimated in the overall immunocompromised post-transplant setting. Further studies, ideally prospective trials with new agents including daratumumab, and thorough assessment of AIC within the existing transplant registries will be crucial to investigate the safety and efficacy profile of novel treatment options such as daratumumab to improve and personalize treatment for patients suffering from this rare complication.

## Data Availability

The datasets used and/or analyzed during the current study are available from the corresponding author on reasonable request.

## Abbreviations

AIC: Immune-mediated cytopenia (allo- or autoimmune cytopenia)
AIHA: Allo- or autoimmune hemolytic anemia
AIT: Allo- or autoimmune thrombocytopenia
C5: Complement factor five
CD38: Cluster of differentiation 38 (cyclic ADP ribose hydrolase)
CD40L: Cluster of differentiation 40 ligand, also called cluster of differentiation154
CMV: Cytomegalovirus
CTLA-4: Cytotoxic T-lymphocyte-associated protein 4
ES: Evans syndrome
GvHD: Graft-versus-host disease
HLA: Human leukocyte antigen
HSCT: Hematopoietic stem cell transplantation
IVIG: Intravenous immunoglobulin G
LRBA: Lipopolysaccharide-responsive and beige-like anchor protein
PBSC: Peripheral blood stem cells
PRCA: Pure red cell aplasia

## References

[1] Gamez-Diaz L, August D, Stepensky P, Revel-Vilk S, Seidel MG, Noriko M (2016). The extended phenotype of LPS-responsive beige-like anchor protein (LRBA) deficiency. J Allergy Clin Immunol..

[2] Jodele S, Davies SM, Lane A, Khoury J, Dandoy C, Goebel J, Myers K, Grimley M, Bleesing J, el-Bietar J, Wallace G, Chima RS, Paff Z, Laskin BL (2014). Diagnostic and risk criteria for HSCT-associated thrombotic microangiopathy: a study in children and young adults. Blood..

[3] Winkelstein JA, Marino MC, Ochs H, Fuleihan R, Scholl PR, Geha R, Stiehm ER, Conley ME (2003). The X-linked hyper-IgM syndrome: clinical and immunologic features of 79 patients. Medicine (Baltimore)..

[4] Ferrua F, Galimberti S, Courteille V, Slatter MA, Booth C, Moshous D, Neven B, Blanche S, Cavazzana M, Laberko A, Shcherbina A, Balashov D, Soncini E, Porta F, al-Mousa H, al-Saud B, al-Dhekri H, Arnaout R, Formankova R, Bertrand Y, Lange A, Smart J, Wolska-Kusnierz B, Aquino VM, Dvorak CC, Fasth A, Fouyssac F, Heilmann C, Hoenig M, Schuetz C, Kelečić J, Bredius RGM, Lankester AC, Lindemans CA, Suarez F, Sullivan KE, Albert MH, Kałwak K, Barlogis V, Bhatia M, Bordon V, Czogala W, Alonso L, Dogu F, Gozdzik J, Ikinciogullari A, Kriván G, Ljungman P, Meyts I, Mustillo P, Smith AR, Speckmann C, Sundin M, Keogh SJ, Shaw PJ, Boelens JJ, Schulz AS, Sedlacek P, Veys P, Mahlaoui N, Janda A, Davies EG, Fischer A, Cowan MJ, Gennery AR (2019). Hematopoietic stem cell transplantation for CD40 ligand deficiency: results from an EBMT/ESID-IEWP-SCETIDE-PIDTC study. J Allergy Clin Immunol..

[5] Even-Or E, Naser Eddin A, Shadur B, Dinur Schejter Y, Najajreh M, Zelig O, Zaidman I, & Stepensky P (2020) Successful treatment with daratumumab for post-HSCT refractory hemolytic anemia. Pediatr blood & cancer, 67(1):e28010. 10.1002/pbc.28010. 10.1002/pbc.28010 31544339

[6] Migdady Y, Ediriwickrema A, Jackson RP, Kadi W, Gupta R, Socola F, Arai S, Martin BA (2020). Successful treatment of thrombocytopenia with daratumumab after allogeneic transplant: a case report and literature review. Blood Adv..

[7] Schuetz C, Hoenig M, Moshous D, Weinstock C, Castelle M, Bendavid M, Shimano K, Tolbert V, Schulz AS, Dvorak CC (2018). Daratumumab in life-threatening autoimmune hemolytic anemia following hematopoietic stem cell transplantation. Blood Adv..

[8] Blennerhassett R, Sudini L, Gottlieb D, Bhattacharyya A (2019) Post-allogeneic transplant Evans syndrome successfully treated with daratumumab. Br J Haematol 187(2):e48–e51. 10.1111/bjh.16171 10.1111/bjh.16171 31441030

[9] Chapuy CI, Kaufman RM, Alyea EP, Connors JM (2018). Daratumumab for delayed red-cell engraftment after allogeneic transplantation. N Engl J Med..

[10] Bathini S, Holtzman NG, Koka R, Singh Z, Wilding E, Zou Y, Ruehle K, Kocoglu MH, Badros A, Hardy N, Yared J, Rapoport AP, Fontaine M, Emadi A, el Chaer F, Dahiya S (2019). Refractory postallogeneic stem cell transplant pure red cell aplasia in remission after treatment with daratumumab. Am J Hematol..

[11] Rautenberg C, Kaivers J, Germing U, Haas R, Ackerstaff S, Hoffmann T, Kobbe G, Schroeder T (2020). Daratumumab for treatment of pure red cell aplasia after allogeneic stem cell transplantation. Bone Marrow Transplant..

[12] Salas MQ, Alahmari A, Lipton JH (2020). Successful treatment of refractory red cell aplasia after allogeneic hematopoietic cell transplantation with daratumumab. Eur J Haematol..

[13] Neunert CE, Despotovic JM (2019). Autoimmune hemolytic anemia and immune thrombocytopenia following hematopoietic stem cell transplant: a critical review of the literature. Pediatr Blood Cancer..

[14] Kruizinga MD, van Tol MJD, Bekker V, Netelenbos T, Smiers FJ, Bresters D, Jansen-Hoogendijk AM, van Ostaijen-ten Dam MM, Kollen WJW, Zwaginga JJ, Lankester AC, Bredius RGM (2018). Risk factors, treatment, and immune dysregulation in autoimmune cytopenia after allogeneic hematopoietic stem cell transplantation in pediatric patients. Biol Blood Marrow Transplant..

[15] Barcellini W, Fattizzo B, Zaninoni A (2019). Management of refractory autoimmune hemolytic anemia after allogeneic hematopoietic stem cell transplantation: current perspectives. J Blood Med..

[16] O’Brien TA, Eastlund T, Peters C, Neglia JP, Defor T, Ramsay NK (2004). Autoimmune haemolytic anaemia complicating haematopoietic cell transplantation in paediatric patients: high incidence and significant mortality in unrelated donor transplants for non-malignant diseases. Br J Haematol..

[17] Faraci M, Zecca M, Pillon M, Rovelli A, Menconi MC, Ripaldi M, Fagioli F, Rabusin M, Ziino O, Lanino E, Locatelli F, Daikeler T, Prete A, Italian Association of Paediatric Haematology and Oncology (2014). Autoimmune hematological diseases after allogeneic hematopoietic stem cell transplantation in children: an Italian multicenter experience. Biol Blood Marrow Transplant..

[18] Wang M, Wang W, Abeywardane A, Adikarama M, McLornan D, Raj K, de Lavallade H, Devereux S, Mufti GJ, Pagliuca A, Potter VT, Mijovic A (2015) Autoimmune hemolytic anemia after allogeneic hematopoietic stem cell transplantation: analysis of 533 adult patients who underwent transplantation at King’s College Hospital. Biol Blood Marrow Transplant. 21(1):60–66. 10.1016/j.bbmt.2014.09.009 10.1016/j.bbmt.2014.09.009 25262883

[19] Lum SH, Selvarajah S, Deya-Martinez A, McNaughton P, Sobh A, Waugh S, Burton-Fanning S, Newton L, Gandy J, Nademi Z, Owens S, Williams E, Emonts M, Flood T, Cant A, Abinun M, Hambleton S, Gennery AR, Slatter M (2020). Outcome of autoimmune cytopenia after hematopoietic cell transplantation in primary immunodeficiency. J Allergy Clin Immunol..

[20] Mahévas M, Michel M, Weill J-C, Reynaud C-A (2013) Long-lived plasma cells in autoimmunity: lessons from B-cell depleting therapy. Front Immunol 4. 10.3389/fimmu.2013.00494 10.3389/fimmu.2013.00494 PMC3873528 24409184

[21] Neely JA, Dvorak CC, Pantell MS, Melton A, Huang JN, Shimano KA (2019). Autoimmune cytopenias in pediatric hematopoietic cell transplant patients. Front Pediatr..

[22] Dalton JE, Bolen SD, Mascha EJ (2016). Publication bias: the elephant in the review. Anesth Analg..

[23] Barcellini W, Fattizzo B, Zaninoni A (2018). Current and emerging treatment options for autoimmune hemolytic anemia. Expert Rev Clin Immunol..

[24] Nahi H, Chrobok M, Gran C, Lund J, Gruber A, Gahrton G, Ljungman P, Wagner AK, Alici E (2019). Infectious complications and NK cell depletion following daratumumab treatment of multiple myeloma. PLoS One..

